# In Vitro and In Vivo Activities of Ruthenium(II) Phosphine/Diimine/Picolinate Complexes (SCAR) against *Mycobacterium tuberculosis*

**DOI:** 10.1371/journal.pone.0064242

**Published:** 2013-05-28

**Authors:** Fernando R. Pavan, Gustavo V. Poelhsitz, Lucas V. P. da Cunha, Marilia I. F. Barbosa, Sergio R. A. Leite, Alzir A. Batista, Sang H. Cho, Scott G. Franzblau, Mariana S. de Camargo, Flávia A. Resende, Eliana A. Varanda, Clarice Q. F. Leite

**Affiliations:** 1 Department of Biological Sciences, College of Pharmacy, Univ Estadual Paulista, Araraquara, São Paulo, Brazil; 2 Chemistry Institute, Univ Federal de Uberlândia, Uberlândia, Minas Gerais, Brazil; 3 Department of Chemistry, Univ Federal de São Carlos, São Carlos, São Paulo, Brazil; 4 Chemistry Institute, Univ Estadual Paulista, Araraquara, São Paulo, Brazil; 5 Institute for Tuberculosis Research, College of Pharmacy, University of Illinois at Chicago, Chicago, United States of America; Université de Montpellier 2, France

## Abstract

Rifampicin, discovered more than 50 years ago, represents the last novel class of antibiotics introduced for the first-line treatment of tuberculosis. Drugs in this class form part of a 6-month regimen that is ineffective against MDR and XDR TB, and incompatible with many antiretroviral drugs. Investments in R&D strategies have increased substantially in the last decades. However, the number of new drugs approved by drug regulatory agencies worldwide does not increase correspondingly. Ruthenium complexes (**SCAR**) have been tested in our laboratory and showed promising activity against *Mycobacterium tuberculosis*. These complexes showed up to 150 times higher activity against MTB than its organic molecule without the metal (free ligand), with low cytotoxicity and high selectivity. In this study, promising results inspired us to seek a better understanding of the biological activity of these complexes. The *in vitro* biological results obtained with the **SCAR** compounds were extremely promising, comparable to or better than those for first-line drugs and drugs in development. Moreover, **SCAR 1** and **4**, which presented low acute toxicity, were assessed by Ames test, and results demonstrated absence of mutagenicity.

## Introduction

Tuberculosis (TB) is an airborne infectious disease caused by *Mycobacterium tuberculosis* (MTB). Mortality rates decreased globally in 2007, with 1.3 million HIV-negative TB patients dying in 2007 and 456,000 deaths occurring amongst individuals who were infected with both TB and HIV [1], [2]. However, multidrug-resistant TB (MDR-TB), extensively drug-resistant TB (XDR-TB), and TB/HIV are stifling attempts to control TB and causing suffering, death and impoverishment worldwide [3].

Additionally, around a third of the world population is infected with MTB in its latent phase and serves as a reservoir for active TB. The purpose of treating latent TB infection is to prevent the development of active disease in high-risk populations, such as people who have had recent contact with individuals with active bacillary TB, or with HIV-positive individuals [4], [5].

Rifampicin (RMP), discovered more than 50 years ago, is a member of the last novel class of antibiotics introduced for the first-line treatment of TB. Drugs in this class are part of a 6 month regimen that is ineffective against MDR and XDR-TB and incompatible with many antiretroviral drugs [6].

The lack of new anti-TB drugs, together with the failure of the current treatments against MDR and XDR-TB, have caused the arising and awakening of many research groups, formed to develop new strategies to identify new drugs against TB [7]. The investments in these research and development (R&D) initiatives have increased substantially in the last decades, but, the number of new drugs approved by drug regulatory agencies has not increased in proportion [8]. Some authors have suggested that research on new drugs, which has almost exclusively used a target-based approach, could be the main determining factor for the low productivity in R&D in recent years [9].

Among these research efforts, Medicinal Inorganic Chemistry has shown a great potential to overcome the problems of TB therapy. It is well known that many metallic elements play a crucial role in living systems. Whereas metal ions are electron-deficient, most biological molecules, such as proteins and DNA, are electron-rich. The attraction of these opposing charges leads to a general tendency for metal ions to bind to and interact with biological molecules [10]. Structure-activity relationships (SARs) studies show that not only does metal complexation enhance the antimicrobial activity of the ligands, but metal itself plays an important part [11], [12].

**Figure 1:**
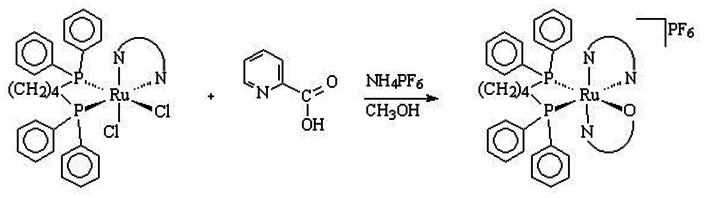
Synthesis of the ruthenium (II) compounds containing the pic ligand.

In previous publications we have shown the synthesis [12] (**Figure 1**) and the potential of ruthenium complexes against TB [11]–[13]. In order to gain a better understanding of the biological activity of these complexes, we have performed further biological studies, reported herein.

## Materials and Methods

### Compounds

The ruthenium complexes [Ru(pic)(dppb)(bipy)]PF_6_ **(SCAR1)**, [Ru(pic)(dppb)(Me-bipy)]PF_6_ **(SCAR2)**_,_ [Ru(pic)(dppb)(phen)]PF_6_ **(SCAR4)**_,_ *cis*-[Ru(pic)(dppe)_2_]PF_6_ **(SCAR5)**_,_ *cis*-[RuCl_2_(dppb)(bipy)] **(SCAR6)** and [Ru(pic)(dppe)(phen)]PF_6_ **(SCAR7– synthesis data not published)** were synthesized at São Carlos Federal University, São Carlos (SP/Brazil), as detailed in previous publications [11], [12], [14].

### Determination of *in vitro* Activity Against MTB H_37_Rv and Standard Drug-resistant Variants

Anti-MTB activity was determined against: H_37_Rv (ATCC 27294), H_37_Rv-rifampicin-resistant (ATCC 35838), H_37_Rv-isoniazid-resistant (ATCC 35822), H_37_Rv-streptomycin-resistant (ATCC 35820) and H_37_Rv-kanamycin-resistant (35827) standard strains, by means of the microplate Alamar Blue assay (MABA) [15]. Stock solutions of the test compounds were prepared in dimethyl sulfoxide (DMSO) (Sigma®) and diluted in Middlebrook 7H9 broth (Difco^tm^), supplemented with oleic acid, albumin, dextrose and catalase (OADC) (BBL/BD^tm^), to obtain a medium at pH 6.8, final drug concentrations ranging from 0.078 to 10 µM. The fluorescence was read (530 nm excitation filter and 590 nm emission filter) in a Victor 2 multi-label reader (Perkin Elmer®) and MIC was defined as the lowest concentration resulting in 90% inhibition of growth of MTB [12]. For MTB H_37_Rv (ATCC 27294), the test was extended to three other conditions: Culture medium at pH 6; 4% Bovine Serum Albumin (BSA) (Sigma®) and 10% Fetal Bovine Serum (FBS) (Sigma®). To standardize the assay, the MICs of RMP, isoniazid (INH), streptomycin (SM) and kanamycin (KM) (Sigma®) were determined on each plate. Each test was set up in triplicate.

### Determination of *in vitro* Activity Against Clinical Isolates of MTB

Twenty-five isolates of MTB (susceptible, mono-resistant and MDR) were obtained from patients with pulmonary TB attending the Clemente Ferreira Hospital in São Paulo City, Brazil, and phenotypic and genotypic resistance to the drugs was determined [16]. The activity of the compounds against these isolates was determined by the Resazurin Microtiter Assay (REMA) [17].

### Determination of *in vitro* Activity Against Non-replicating Persistent MTB H_37_Rv

The potential activity against non-replicating persistent (NRP) MTB was assessed by Low Oxygen Recovery Assay (LORA) [18]. MTB H_37_Rv, containing the pFCA-luxAB plasmid, which synthesizes luciferase, was employed in this method [19], [20]. To obtain the bacillus in the latent phase (NRP II), Wayne’s model was used [21]–[23]. Microplate cultures were placed under anaerobic conditions (oxygen <0.16%) by using an Anoxomat Model WS-8080, with two cycles of evacuation and filling with a mixture of 10% H_2_, 5% CO_2_, balance N_2_. Plates were incubated at 37°C for 10 days and then transferred to a normal atmosphere, enriched with 5% CO_2_, incubated for 28 h to “recover” [18]. A 1% solution of n-decanal in ethanol was freshly diluted 10-fold in PBS and 100 µL added to each well. Luminescence was measured in a Victor 2 multi-label reader (1 s reading time). Compounds reducing viability under these NRP conditions led to a decreased luciferase signal following aerobic recovery. LORA-MIC was defined as the lowest concentration, resulting in 90% inhibition of growth of MTB [18]. Each test was set up in triplicate.

### Checkerboard Synergy Assay

**Figure 2:**
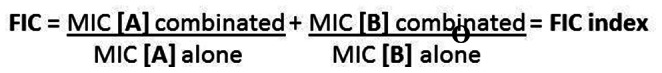
Calculation of Fraction Inhibitory Concentration (FIC).

Interactions between ruthenium (Ru) complexes and first-line drugs (namely: INH, RMP, SM, ethambutol (EMB) and moxifloxacin (MOX)) were assayed against MTB H_37_Rv (ATCC 27294), as described by Moody (1992) [24] with some modifications described by Luna Herrera et al. (2007) [25]. For this assay, combinations of two compounds (2D) (e.g. Compound (1) and isoniazid) were tested in a 96-well microtiter plate (NUNC^tm^), where the test compound (A) was transferred to row A (X axis) of columns 2–9 at a concentration four times higher than its MIC alone, after which two-fold serial dilutions were made to row H. Compound (B) (first-line drug) was then transferred to column 2 (Y axis) of rows A-H at a concentration four times higher than its MIC alone, after two-fold dilutions were made to column 8. Column 10 was used as a positive control (bacterial growth) and column 11 as a negative control (only media). The MICs for the compounds A and B alone were assessed every time an assay was performed. After the checkerboard of drug combinations was set up in the wells, MTB H_37_Rv (ATCC 27294) was thawed and added, yielding a final testing volume of 200 µL with 2×10^4^ CFU/mL. Microplates were incubated for 7 days at 37°C, after which a mixture of Alamar Blue solution (20 µL) and sterile 10% Tween 80 (12 µL) was added. The fluorescence was read (530 nm excitation filter and 590 nm emission filter) in a Victor 2 multi-label reader. Results were analyzed in terms of Fraction Inhibitory Concentration (FIC) (**Figure 2**) [24]. In each row, a certain concentration of B was the lowest causing 90% inhibition of growth (MIC [B]), while in each column, one concentration of A was identified as MIC A. A diagonal line across the plate joined wells containing both MIC [A] (for the column) and MIC [B] (for the row). Considering these values as “MIC combined”, FIC was calculated for each of these wells and the lowest FIC was interpreted as follows: ≤1 synergism; >1–4 indifferent; >4 antagonism [26]. Each test was set up in triplicate.

### Activity Spectrum Assay

Antimicrobial assays were performed against *Staphylococcus aureus* (ATCC 29213), *Escherichia coli* (ATCC 25922), *Candida albicans* (ATCC 10231) and *Mycobacterium smegmatis* luxAB (MC^2^155). All microorganisms strains were grown until log phase in various culture media: 16–20 hours in cation-adjusted Mueller Hinton broth (CAMH) (Difco^tm^) for *S. aureus* and *E. coli*; 24–48 hours in RPMI-1640 (ATCC^ tm^) for *C. albicans* and 48–72 hours in Middlebrook 7H9 broth supplemented with OADC enrichment and kanamycin (20 µg/mL) for *M. smegmatis.* Cultures were divided into aliquots and frozen at −80°C. *S. aureus*, *E. coli* and *C. albicans* culture suspensions were prepared and their turbidities matched to the optical density of the McFarland no. 0.5 standard. These were diluted further to 5×10^5^ CFU/mL for *S. aureus* and *E. coli* and 1.5×10^6^ for *C. albicans* **;** 100 µL of each suspension was then transferred to microplate wells, together with serial dilutions of the test compounds. After incubation at 37°C for 24 hs (*S. aureus* and *E. coli*) and 48 hs (*C. albicans*), the absorbance was determined at 600 nm and the MIC of each compound was calculated. The activity against *M. smegmatis* was determined by MABA [15] and luminescence [18] assays after 72 hours incubation at 37°C. All assays were read in a Victor 2 multi-label reader. Each test was set up in triplicate.

### Acute Oral Toxicity

The safety profiles of six Ru complexes were assessed by means of acute toxicological assay in mice. For this purpose, female C57BL/6 mice (18 to 20 g), aged 4–6 weeks, were used. The animals were housed in groups of six and maintained under controlled temperature (22±2°C) and relative humidity (60–70%), and a 12 h light-dark cycle. Food and water were freely available. The experiments reported herein were performed in accordance with OECD 2001 [27]. The Research Ethics Committee of UNESP/FCFAR approved all the experimental procedures under resolution 41/2008. The number of animals was the minimum necessary to demonstrate consistent effects for drug treatments. A single dose of the compound suspended in 0.5% carboxymethylcellulose (CMC/Sigma®) (2000 mg/kg body weight) was orally administered to every six mice (six mice/compound). When this concentration caused more than 50% deaths, another lower concentration was administered (1000 and 500 mg/kg body weight). Control animals received the vehicle diluted into water (0.5% CMC) by the oral route. Clinical symptoms, including death, were observed in the first 30 min, 1 hour, 2 hours, 4 hours, 6 hours, 12 hours and 24 hours and then once a day for 14 days. Body weight was measured at the beginning and the end of experiments. At the end of the experimental period, the animals were euthanized and various organs (namely heart, spleen, liver and kidneys) were carefully dissected and their absolute weights were determined.

### Mutagenicity

Mutagenic activity was evaluated by the Salmonella/microsome assay, using the *Salmonella typhimurium* tester strains TA98, TA100, TA97a and TA102, kindly provided by Dr. B.N. Ames (Berkeley, CA, USA), with (+S9) and without (−S9) metabolization, by the pre-incubation method [28]. The strains were grown from frozen cultures overnight for 12–14 h in Oxoid Nutrient Broth No. 2. The metabolic activation mixture (S9 fraction), prepared from livers of Sprague–Dawley rats treated with the polychlorinated biphenyl mixture Aroclor 1254 (500 mg/kg), was purchased from Molecular Toxicology Inc. (Boone, NC, USA) and freshly prepared before each test. The metabolic activation system consisted of 4% S9 fraction, 1% 0.4 M MgCl 2, 1% 1.65 M KCl, 0.5% 1 M D-glucose-6-phosphate disodium and 4% 0.1 M NADP, 50% 0.2 M phosphate buffer and 39.5% sterile distilled water [28].

For the determination of the mutagenic activity, five different concentrations of **SCAR1** and **4** (6.25–150 µg/ plate), diluted in DMSO, were assayed. The concentrations of the compounds were selected on the basis of a preliminary toxicity test. In all subsequent assays, the upper limit of the dose range tested was either the highest non-toxic dose or the lowest toxic dose determined in this preliminary assay. Toxicity was detected either as a reduction in the number of histidine revertants (His+), or as a thinning of the auxotrophic background (i.e., background lawn).

The various concentrations of the compounds to be tested were added to 0.5 Ml of 0.2 M phosphate buffer, or to 0.5 Ml of 4% S9 mixture, with 0.1 Ml of bacterial culture and then incubated at 37°C for 20–30 min. Next, 2 Ml of top agar was added and the mixture poured on to a plate containing minimal agar.

The plates were incubated at 37°C for 48 h and His+ revertant colonies were counted manually. All experiments were analyzed in triplicate. Results were analyzed with the statistical software package Salanal 1.0 (U.S. Environmental Protection Agency, Monitoring Systems Laboratory, Las Vegas, NV, from Research Triangle Institute, RTP, NC, USA), adopting the Bernstein et al. model [29]. Data (revertants/plate) were assessed by analysis of variance (ANOVA), followed by linear regression. The mutagenic index (MI) was also calculated for each concentration tested, this being the average number of revertants per plate with the test compound divided by the average number of revertants per plate with the negative (solvent) control. A test solution was considered mutagenic when a dose–response relationship was detected and a two-fold increase in the number of mutants (MI ≥2) was observed for at least one concentration [30]. The standard mutagens used as positive controls in experiments without S9 mix were 4-nitro-o-phenylenediamine (NOPD) (10 µg/plate) for TA98 and TA97a, SA (1.25 µg/plate) for TA100 and mitomycin C (MMC) (0.5 µg/plate) for TA102. In experiments with S9 activation, 2-amino-anthracene (2-AA) (1.25 µg/plate) was used with TA98, TA97a and TA100 and 2-amino-fluorene (2-AF) (10 µg/plate) with TA102. DMSO (50 Μl/plate) served as the negative (solvent) control.

## Results

### Anti-MTB Activity Against Susceptible and Drug-resistant Strains

**Table 1** shows the MICs of the **SCAR** compounds assayed against MTB H_37_Rv under various conditions and against standard drug-resistant strains under normal conditions.

**Table 1:** MICs of the **SCAR** compounds acting on MTB H_37_Rv under various conditions and on drug-resistant variants under normal conditions.

Compound	MIC (µM)							
	Normal	pH 6	Protein Binding		Drug Resistant Variants*			
			4% BSA	10% FBS	rRMP	rINH	rSM	rKM
SCAR1	1.2	2.4	2.5	1.3	2.6	2.5	3.3	2.5
SCAR2	1.2	2.2	2.0	1.3	3.1	2.5	3.3	2.5
SCAR4	1.4	2.7	3.5	1.9	4.2	2.8	4.2	2.9
SCAR5	0.8	1.7	1.7	0.8	1.8	1.9	1.9	2.0
SCAR6	1.6	4.9	3.2	3.1	5.0	6.3	6.6	6.6
SCAR7	2.1	2.7	2.8	2.5	5.0	4.0	5.4	5.4
**Standard Drug**								
RMP	0.1	0.2	0.1	0.1	>2	n.d.	n.d.	n.d.
INH	0.1	0.2	0.3	0.2	n.d.	>4	n.d.	n.d.
SM	0.5	0.8	0.9	1.3	n.d.	n.d.	>7.9	n.d.
KM	4.1	n.d.	n.d.	n.d.	n.d.	n.d.	n.d.	>10.2

Normal conditions – those recommended by the manufacturer (pH = 6.8).

* rRMP - H_37_Rv -rifampicin-resistant (ATCC 35838).

* rINH - H_37_Rv-isoniazid-resistant (ATCC 35822).

* rSM - H_37_Rv-streptomycin-resistant (ATCC 35820).

* rKM - H_37_Rv -kanamycin-resistant (ATCC 35827).

n.d. - not determined.

The MICs of the **SCAR** compounds in normal conditions were reassessed by a second method (MABA) using the same criteria as in the first method (REMA) [15], [17] and the results obtained earlier [11], [12] were the same as in this study, with no significant difference (data not shown). In fact, the reaction used in both methods is the same.

The compounds and drugs were also exposed to acid conditions and their MICs determined (**Table 1**). The ratio MIC_pH6,0_/MIC_normal_ for the **SCAR** compounds ranged from 1.3 to 3.1, while for the standard drugs it ranged from 1.6 to 2.0.

MICs were also determined under two more conditions: with 4% BSA and 10% FBS in the medium. The BSA and FBS concentrations used in this assay were previously determined as the highest concentrations causing no bacterial inhibition. Results are shown in **Table 1**. The ratios MIC_4%BSA_/MIC_normal_ and MIC_10%FBS_/MIC_normal_ for the test compounds ranged from 1.3 to 2.7 and 1.0 to 1.9, respectively, and for the standard drugs from 1.8 to 3.0 and 2.0 to 2.6, respectively.

As part of our development pipeline, the **SCAR** compounds were also tested against variant strains resistant to four standard drugs (**Table 1**) under normal culture conditions. According to **Table 1**, all **SCAR** compounds showed activity against these strains. **SCAR6** was less active than the other compounds, with a MIC_resistant_/MIC_normal_ ratio ranging from 3.1 to 4.1, while the others had ratios lower than 3. However, **SCAR6** may still be considered active against drug-resistant variants.

### Anti-MTB Activity Against Clinical Isolates

In order to confirm the activity of SCAR against drug-resistant strains, the compounds were tested on twenty-five clinical isolates. Results are presented in **Table 2**.

**Table 2:** MICs of the **SCAR** compounds acting on susceptible, mono-resistant and MDR clinical isolates.

Isolate/strain no.	Standard Drug BACTEC™ MGIT™ 960				SCAR compound (REMA) - µM					
	RMP	INH	SM	EMB	1	2	4	5	6	7
**H37Rv**	S	S	S	S	1.2	1.2	1.4	0.8	1.6	2.1
**Susceptible**										
**16**	S	S	S	S	6.5	3.2	3.7	2.7	4.1	3.6
**40**	S	S	S	S	1.6	1.6	1.8	1.3	16.6	nd
**48**	S	S	S	S	13.1	3.2	3.7	1.3	>33.1	nd
**66**	S	S	S	S	>26.1	3.2	7.3	2.7	>33.1	nd
**68**	S	S	S	S	3.3	1.6	1.8	1.3	2.1	nd
**71**	S	S	S	S	3.3	1.6	1.8	1.3	>33.1	nd
**72**	S	S	S	S	3.3	3.2	3.7	1.3	>33.1	nd
**75**	S	S	S	S	26.1	1.6	1.8	1.3	>33.1	nd
**Mono-Resistant**										
**15**	S	**R**	S	S	6.5	1.6	3.7	1.3	4.1	3.6
**77**	S	**R**	S	S	6.5	6.4	7.3	5.3	nd	7.2
**98**	**R**	S	S	S	1.6	1.6	1.8	0.3	16.6	1.8
**181**	S	S	**R**	S	1.6	1.6	1.8	0.3	nd	1.8
**MDR**										
**84**	**R**	**R**	S	S	3.3	3.2	1.8	1.3	nd	1.8
**145**	**R**	**R**	S	S	1.6	1.6	1.8	0.7	nd	1.8
**173**	**R**	**R**	S	S	3.3	3.2	3.7	2.7	>33.1	3.6
**176**	**R**	**R**	S	S	26.1	1.6	3.7	2.7	>33.1	nd
**46**	**R**	**R**	**R**	S	6.5	1.6	3.7	2.7	16.6	7.15
**142**	**R**	**R**	**R**	S	26.1	1.6	1.8	2.7	4.1	nd
**92**	**R**	**R**	**R**	**S**	>26.1	6.4	7.3	5.3	>33.1	nd
**93**	**R**	**R**	**R**	**S**	26.1	3.2	3.7	2.7	>33.1	nd
**59**	**R**	**R**	**S**	**R**	13.1	1.6	1.8	0.3	>33.1	nd
**61**	**R**	**R**	**R**	**R**	6.5	1.6	3.7	1.3	>33.1	7.15
**97**	**R**	**R**	**R**	**R**	13.1	3.2	1.8	1.3	33.1	nd
**104**	**R**	**R**	**R**	**R**	3.3	0.8	1.8	0.3	8.3	0.9
**185**	**R**	**R**	**R**	**R**	25	0.39	0.39	039	>25	nd

S: Susceptible; R: Resistant; n.d.: not determined.

Thus, eight isolates were susceptible to all drugs, 4 mono-resistant and 13 MDR-TB, according to the technique of BACTEC™ MGIT™ 960 technique. Applying the criterion that compounds with MIC ≤10 µM are potential anti-TB agents [7], we may summarize the results as follows:

**SCAR1** showed promising activity (MIC ≤10 µM) on 15 isolates (5 susceptible, 4 mono-resistant and 6 MDR-TB), with MICs ranging from 1.6 to 6.5 µM. For the resistant strains (MIC >10 µM), the MIC ranged from 13.1 to >26.1 µM.

**SCAR2, 4, 5 and 7** showed promising activity on all isolates. The range of MIC values varies from 0.39 to 6.4; 0.39 to 7.3; 0.3 to 5.3; 0.9 to 7.2 µM, respectively.

**SCAR6** showed promising activity on only 4 isolates (2 susceptible, 1 mono-resistant and 1 MDR-TB) among the 20 analyzed, with MICs ranging from 2.1 to 8.3 µM. Regarding resistant strains, the MIC ranged from 16.6 to >33.1 µM.

### Anti-MTB Activity Against Non-replicating Persistent Bacteria

**Table 3** shows the MICs of the **SCAR** compounds acting on MTB H_37_Rv (pFCA-luxAB) in the latent phase.

**Table 3:** MIC of the **SCAR** compounds acting on non-replicating persistent (NRPII) MTB H_37_Rv.

Compound	LORA (µM)
SCAR1	0.55
SCAR2	0.46
SCAR4	0.53
SCAR5	0.31
SCAR6	0.42
SCAR7	1.21
**Standard Drug**	
RMP	0.84
INH	>507
SM	3.52
EMB	>24.2
MOX	6

The NRPII MICs of the **SCAR** compounds were smaller than those measured under aerobic conditions (**Table 1**). Their activity under these conditions was better than the drugs currently used to treat TB (RMP, INH, EMB and SM) and MOX. They were also more effective than a drug currently undergoing clinical development, PA-824 [31].

### Checkerboard Synergy

**Table 4** shows the interactions detected between **SCAR** compounds and the standard drugs.

**Table 4:** Interaction results of the **SCAR** compounds with INH, RMP, SM, EMB and MOX against MTB H_37_Rv.

Standard Drug/Compound	FIC	Interaction	Standard Drug/Compound	FIC	Interaction
**RMP**			**EMB**		
SCAR1	1.0	Synergism	SCAR1	1.6	Indifferent
SCAR2	2.0	Indifferent	SCAR2	1.6	Indifferent
SCAR4	1.0	Synergism	SCAR4	3.3	Indifferent
SCAR5	1.1	Indifferent	SCAR5	1.7	Indifferent
SCAR6	1.0	Synergism	SCAR6	1.6	Indifferent
SCAR7	1.0	Synergism	SCAR7	2.4	Indifferent
**INH**			**MOX**		
SCAR1	1.4	Indifferent	SCAR1	2.5	Indifferent
SCAR2	1.0	Synergism	SCAR2	1.3	Indifferent
SCAR4	1.0	Synergism	SCAR4	1.3	Indifferent
SCAR5	1.0	Synergism	SCAR5	2.6	Indifferent
SCAR6	0.5	Synergism	SCAR6	0.8	Synergism
SCAR7	1.0	Synergism	SCAR7	2.5	Indifferent
**SM**					
SCAR1	1.3	Indifferent			
SCAR2	1.3	Indifferent			
SCAR4	1.3	Indifferent			
SCAR5	1.4	Indifferent			
SCAR6	0.5	Synergism			
SCAR7	1.3	Indifferent			

All **SCAR** compounds (**Table 4**) showed synergistic interaction with some of the drugs analyzed. Results ranged from synergism (when the activity of the compound is enhanced or potentiated by the other drug that is tested together) and indifferent (when the activity of both remained the same, without mutual interference). The best overall synergistic interactions were observed with INH. The only exception in this case is the compound **SCAR1** (indifferent). The only drug that tested indifferent to all SCAR was EMB.

### Spectrum of Activity

**Table 5** shows the activities of the **SCAR** compounds and the standard drugs.

**Table 5:** Spectrum of activity of the SCAR compounds.

Compound	MIC (µM)			
	*E. coli*	*S. aureus*	*C. albicans*	*M. smegmatis*
SCAR1	>10.4	5.1	>10.4	5.2
SCAR2	>10.2	2.4	>10.2	5.0
SCAR4	>11.8	4.6	>11.8	5.7
SCAR5	>8.5	0.3	1.9	5.3
SCAR6	>13.3	5.9	>13.3	>13.3
SCAR7	>11.4	5.5	>11.4	5.6
**Standard drug**				
GTM	1.0	0.6	n.d.	n.d.
RMP	n.d.	n.d.	n.d.	>100
INH	n.d.	n.d.	n.d.	>100
SM	n.d.	n.d.	n.d.	0.9
MOX	n.d.	n.d.	n.d.	0.2
MET	n.d.	n.d.	n.d.	>512

n.d. – not determined.

With exception to **SCAR6**, the compounds that were active against MTB also showed activity against *M. smegmatis*. This activity was lower than that shown by SM and MOX (MICs 0.9 and 0.2 µM respectively), but higher (by more than 100x) than that of RMP, INH and MET. In relation to *S. aureus*, all the compounds showed inhibitory activity similar to that against *M. smegmatis*. **SCAR2** and **SCAR5** (2.4 and 0.3 µM respectively) were the most active against *S. aureus* and **SCAR5** showed similar activity to that of gentamicin (GTM) (0.6 µM). Only **SCAR5** was active against *C. albicans* (1.9 µM). Against *E. coli*, none of the compounds showed significant inhibitory activity. In short, the test compounds (except **SCAR6,** which was restricted to MTB and *S. aureus*) were active against gram-positive bacteria and mycobacteria in a test model.

### Acute Oral Toxicity

**Table 6** shows the acute oral toxicity of the **SCAR** compounds and the standard drug RMP.

**Table 6:** Acute oral toxicity of **SCAR** compounds and percentage of losses.

Compound	Dose (mg/kg body weight)	Deaths (%)
SCAR1	2000	0
	1000	n.d.
SCAR2	2000	83.3
	1000	16.6
SCAR4	2000	16.6
	1000	n.d.
SCAR5	2000	50
	1000	n.d.
SCAR6	2000	0
	1000	n.d
SCAR7	2000	100
	1000	83.3
	500	0
RMP	2000	0
	1000	0

n.d. not determined.

The acute toxicity observed was classified into categories as described by the OECD, (2001) [27]. Relevant results are shown below:

**SCAR1, 6 and RMP –** No accidental loss at the concentration of 2000 mg/kg body weight (or at 1000 mg/kg body weight for RMP). Hippocratic screening (behavior) did not show any changes. Macroscopic examination of the organs showed no morphological changes and statistical analysis of the final weights and features of organs showed no significant difference (data not shown). As for categories, **SCAR1** and **6** belong to class 5 (substances with LD_50_ greater than 2000 and less than 5000 mg/kg body weight).

**SCAR4 and 5–** One out of six mice was lost for **SCAR4** and three for **SCAR5**, at the concentration of 2000 mg/kg body weight. According to the hippocratic screening, both caused a rare reduction of general activity in two animals during the study period. Macroscopic examination of the organs showed no morphological changes and statistical analysis of the final weight and organ features showed no significant difference (data not shown). Thus, **SCAR4** and **5** were also placed in class 5.

**SCAR2 and 7**– Five accidental losses were observed for **SCAR2** and six for **SCAR7** at 2000 mg/kg body weight. At 1000 mg/kg body weight **SCAR 2** showed one accidental loss and **SCAR7** five. **SCAR7** was also tested at 500 mg/kg body weight and did not lead to any loss. Hippocratic screening did not show any changes. Macroscopic examination of the organs showed significant statistical differences in the final weight, hepatosomatic index and pancreas: body weight ratio, for both compounds (data not shown). **SCAR2** and **7** were classified in class 4.

### Mutagenicity

**Tables 7** and **8** show the mean number of revertants/plate (M), the standard deviation (SD) and the mutagenic index (MI) after treatment with **SCAR1** and **SCAR4**, observed in *S. typhimurium* strains TA98, TA100, TA102 and TA97a, in the presence (+S9) and absence (−S9) of metabolic activation. Mutagenicity assays show that these both compounds did not induce any increase in the number of revertant colonies relative to the negative control, indicating the absence of any mutagenic activity by this method.

**Table 7:** Mutagenic activity expressed as the mean and standard deviation of the number of revertants and mutagenic index (MI) (in brackets) in strains TA98, TA100, TA102 and TA97a exposed to SCAR01 at various doses, with (+S9) or without (-S9) metabolic activation.

Treatment Number of revertants (M ± SD)/plate and MI										
µg/plate		TA 98		TA 100		TA 102		µg/plate	TA 97a	
		−S9	+S9	−S9	+S9	−S9	+S9		−S9	+S9
**SCAR01**	**0.00** a	17±3	40±2	114±12	145±5	352±24	300±21	**0.00** a	107±13	114±15
	**18.8**	19±2 (1.1)	40±4(1.0)	135±15(1.2)	138±10 (0.9)	333±34 (0.9)	338±18 (1.1)	**6.25**	111±10 (1.0)	131±13 (1.2)
	**37.5**	21±2 (1.2)	40±4(1.0)	115±31(1.0)	146±12(1.0)	299±29 (0.8)	283±24 (0.9)	**12.5**	116±21 (1.1)	98±22(0.9)
	**75**	18±3 (1.0)	39±7 (1.0)	125±19(1.1)	145±16 (1.0)	351±52 (1.0)	299±45 (1.0)	**25**	100±17 (0.9)	128±18 (1.1)
	**112.5**	18±4 (1.0)	40±9 (1.0)	127±10 (1.1)	141±10 (1.0)	301±35 (0.9)	323±49 (1.1)	**37.5**	93±12 (0.9)	94±28 (0.8)
	**150**	15±2 (0.9)	37±2 (0.9)	136±13(1.2)	114±19(0.8)	361±42 (1.0)	312±20 (1.0)	**50**	82±19 (0.8)	98±37 (0.9)
	**C +**	797±79^ b^	1204±155^e^	1193±39^c^	1229±94 e	1192±49^d^	1804±43 e	**C +**	716±74^ b^	1636±162 e

a Negative control: dimethylsulfoxide (DMSO - 100 µL/plate); C+ = Positive control -^b^4 -nitro-o-phenylenediamine (NOPD –10.0 µg/plate – TA98, TA97a);

c sodium azide (1.25 µg/plate –TA100);

dc mitomycin (0.5 µg/plate – TA102), in the absence of S9 and

e 2-anthramine (1.25 µg/plate – TA97a, TA98, TA100);

f 2-aminofluorene (10.0 µg/plate – TA102), in the presence of S9.

**Table 8:** Mutagenic activity expressed as the mean and standard deviation of the number of revertants and mutagenic index (MI) (in brackets) in strains TA98, TA100, TA102 and TA97a exposed to SCAR04 at various doses, with (+S9) or without (-S9) metabolic activation.

Treatment Number of revertants (M ± SD)/plate and MI									
µg/plate		TA 98		TA 100		TA 102		TA 97a	
		−S9	+S9	−S9	+S9	−S9	+S9	−S9	+S9
**SCAR04**	**0,00** a	19±4	28±3	138±13	129±8	365±18	350±22	131±11	154±8
	**6.25**	20±2 (1)	34±2 (1.2)	142±17(1.0)	144±13 (1.1)	353±29 (1.0)	378±15 (1.0)	141±9 (1.1)	135±11 (0.8)
	**12.5**	22±2 (1.1)	33±5 (1.2)	149±29(1.1)	132±12 (1.0)	359±19 (1.0)	333±14 (0,9)	129±19 (1.0)	161±20 (1.0)
	**25**	17±3 (0.9)	30±8 (1.1)	131±17(0.9)	123±9 (0.9)	343±48 (0.9)	389±36 (1.1)	135±17 (1.0)	127±19 (0.8)
	**37.5**	17±4 (0.9)	27±7 (1.0)	121±12 (0.9)	147±14 (1,1)	337±31 (0.9)	299±38 (0.8)	120±12 (0.9)	121±10 (0.8)
	**50**	16±2 (0.8)	29±5 (1.0)	142±16(1.0)	120±17(0.9)	361±46 (1.0)	280±24 (0.8)	126±18 (1.0)	140±27 (0.9)
	**C +**	763±68^ b^	1123±117^e^	1112±43^c^	1248±94 e	1212±38^d^	1504±29 e	767±74^ b^	1546±131 e

a Negative control: dimethylsulfoxide (DMSO - 100 µL/plate); C+ = Positive control -^b^4 -nitro-o-phenylenediamine (NOPD –10.0 µg/plate – TA98, TA97a);

c sodium azide (1.25 µg/plate –TA100);

dc mitomycin (0.5 µg/plate – TA102), in the absence of S9 and

e 2-anthramine (1.25 µg/plate – TA97a, TA98, TA100);

f 2-aminofluorene (10.0 µg/plate – TA102), in the presence of S9.

## Discussion

The lack of any new anti-TB drugs for more than 50 years, despite the increased investments in R&D, is extremely disappointing and indicates that new lines of research must be followed [8]. Many groups have been searching for new anti-TB drugs by means of target-based assays or phenotypic assays on whole cells [2], [9]. Our group has sought promising new candidates via a pipeline based on phenotypic assays [7]. Excited by our results with Ru complexes [11]–[13] and encouraged by recent reviews [2], [9], we initiated this study in search of a better biological understanding of these **SCAR** compounds.

The relevance of the anti-MTB assay in acid environment lies in the attempt to mimic conditions in the lungs, whose pH is around 6.0. The pH inside pulmonary TB lesions is also equals to or less than 6.0 [32]. This idea is reinforced when we consider some drugs currently used in TB treatment, such as pyrazinamide (PZA). PZA is a pro-drug with higher MIC and no activity under normal conditions, but PZA is valuable in TB control, because it becomes active in acid conditions and may kill bacteria inside the granuloma, while other drugs lose their activity under these conditions [31], [32]. In microbiology, an activity range until twofold dilutions is totally accepted as no statistical significance. RMP, for example, is described in the literature with a MIC ranging from 0.1 to 0.4 µM under normal conditions (is a twofold dilution) [31]. In conclusion, the current experiments with the **SCAR** compounds showed excellent activity under normal conditions and remained stable and active in mildly acid medium.

The compounds were exposed to two further conditions: 4% BSA and 10% FBS. BSA is similar in physical and chemical properties to human serum albumin, the most abundant plasma protein. FBS is a complex mixture composed of a number of serum constituents, such as albumin, α, β and γ globulins, urea, creatinine and hemoglobin. The i*n vitro* assay in this medium is important as it mimics the conditions in human plasma. An increase in MIC to more than double the normal value indicates an interaction between the test compound and a plasma protein. Under these conditions, the drug will probably not reach its tissue target at an adequate concentration. This experiment showed that plasma proteins do not interfere with the activity of the **SCAR** compounds.

The search for compounds against mycobacterium resistant strains has been intensified in recent years, especially since the emergence of MDR and XDR TB [6]. Some new compounds, active against these strains, have emerged, such as PA-824, TMC-207 and SQ-109 [31]. Currently, these compounds are being subjected to several clinical trials [4]. TMC-207 (“Sirturo®) is the first time a new drug is being introduced specifically for MDR-TB. Regarding the **SCAR** compounds, all showed some activity against the four resistant variants tested. These results suggest that these compounds have a different mechanism of action than the standard drugs tested. In a previous study we have shown, by circular dichroism spectroscopy, that **SCAR1** interacts with DNA, suggesting that the mechanism of its tuberculostatic action be based on such an interaction [12].

However, the activity of **SCAR** compounds on the clinical isolates was not uniform. **SCAR2**, **4**, **5** and **7** were active on all 25 isolates, which included strains sensitive and resistant to the classical drugs tested. But **SCAR1** and **6** were ineffective against some of the isolates, distributed among both the sensitive and resistant strains. The mainly significant difference between **SCAR1** ([Ru(pic)(dppb)(bipy)]PF6 and **6** cis-[RuCl_2_(dppb)(bipy)] and the other complexes is that they have a common organic ligand (bipyridine – bipy), whereas the others do not. The intrinsic resistance of these isolates probably involves this ligand, since the other ligands are common in all complexes. **SCAR1** and **6** have the ligand 2,2′-bipyridine, contrary to **2**, in which bipy is substituted by its dimethylated derivative, and **SCAR4**, **5** and **7**, where this ligand is replaced by others. One hypothesis is that resistant strains have acquired the ability to metabolize all other complexes except those with the bipy.

A feature of *M. tuberculosis* that greatly complicates the control and eradication of TB is its ability to remain dormant for months or years in the host, even under continuous chemotherapy [21]. MTB, which requires plentiful O_2_ for active growth, shifts down to a non-replicating persistent (NRP) state when subjected to gradual depletion of dissolved O_2_. The shift down from active aerobic growth (AAG) during depletion of available O_2_ proceeds in two discrete steps, firstly to microaerophilic NRP stage 1, then to anaerobic NRP stage 2 [22]. A physiological understanding of MTB in the latency state is important to suggest strategies for developing new chemotherapeutic agents able to eradicate such infections [4]. The anti-TB drugs available so far have failed to eradicate the dormant cellular forms [33]. Some new compounds have shown *in vitro* activity against latent infection, such as TMC-207 and PA-824 [4]. This study showed (**Table 3**) that the **SCAR** compounds may hold promise for the treatment of latent infection. The *in vitro* activity against the NRP II stage (LORA model) was higher than the activity under aerobic conditions (**Table 1**) and better than that of the drugs currently used in therapy**:** RIF, INH and EMB (inactive in NRP II stage), SM and MOX and even the compound PA-824, currently in clinical phase of development.

Currently, TB treatment in Brazil is based on fixed combined doses of RMP, INH, EMB and PZA in the first two months and RMP and INH in the following four months, while in other countries the same drugs are taken separately, as suggested by the World Health Organization (WHO) [34]. Any new drug must be used in combination with another, to avoid the appearance of resistant strains. In line with this requirement, the interactions between a new compound and the current anti-TB drugs are assessed through the 2D checkerboard experimental design [24], [25] (**Table 4**). All **SCAR** compounds showed fair results (synergism or indifferent) in the test of interactions with all five drugs analyzed. Results with INH were particularly good (all but one showed synergism).

The activity spectrum defines the selectivity of the drug. As shown in **Table 5**, the **SCAR** compounds (except **SCAR6,** which is restricted to MTB and *S. aureus*) appear to be active in gram-positive bacteria and mycobacteria model.

Acute toxicity describes the adverse effects of a substance, resulting from a single exposure in a short space of time (24 hours). Acute toxicity adverse effects should occur within 14 days of the administration of the substance [27]. The results (**Table 6**) showed that **SCAR1**, **4** and **6** have low toxicity and **SCAR2**, **5** and **7** have medium toxicity. However, although **SCAR 2**, **5** and **7** were more toxic than RMP (**Table 6**), they are less toxic than some second-line drugs such as amikacin and capreomycin [31], denoting their relative therapeutic safety.

According to the results of acute toxicity, the mutagenic assay was performed. The Ames test is used worldwide as an initial screen to determine the mutagenic potential of new chemicals and drugs. This test is also used to furnish data for submission to regulatory agencies, for the registration or acceptance of many chemicals, including drugs and biocides. International guidelines have been developed to be used by corporations and testing laboratories, in order to ensure uniformity of testing procedures [35].

In this study, the mutagenicity of **SCAR1** and **4**, which presented low acute toxicity, was assessed by the Ames test, using different concentrations of the compound and four bacterial strains (*Salmonella typhimurium* TA97a, TA98, TA100 and TA102), each strain carrying different mutations in various genes in the histidine operon. A metabolic activation system (S9 mix) was added to *S. typhimurium* during the assay to metabolize the compounds by cytocrome P450, enzymes extracted from rat liver.

The results of the mutagenicity assay are presented in **Tables 7** and **8** **.** The MI was not higher than 2 at any tested concentration, indicating the absence of mutagenic activity of **SCAR1** and **4**, a positive step towards ensuring its safe use in medicine. Considering the possible use of **SCAR** in tuberculosis treatment, a lack of mutagenic effects in animal cells and bacteria is highly relevant.

### Conclusion

The biological results in the *in vitro* assays of the **SCAR** compounds were extremely promising, comparable to or even better than those of first-line drugs and drugs in development. These compounds responded positively to some of the criteria that a new drug against TB must meet, namely activity in dormant bacteria, activity in resistant bacteria and absence of mutagenicity by Ames test and negative interaction with other anti-TB drugs. However, it is necessary to clarify that all *in vitro* assays have some limitations that cannot be reproduced in humans. However, we believe the experiments chosen for the pipeline are the closest to reality. The molecular study to understand the action mechanism of these compounds is in progress and preliminary results have suggested the activity to be related with cell wall biosynthesis. This is the first time anti-mycobacterial activity has been demonstrated in this class of Ru(II) complexes, which may constitute a new family of anti-TB drugs.
